# Development and evaluation of a mechanical ventilator-sharing system

**DOI:** 10.3389/fmed.2024.1356769

**Published:** 2024-02-16

**Authors:** Satyanarayana Achanta, Michael A. Gentile, Neil R. Euliano

**Affiliations:** ^1^Department of Anesthesiology, Duke University School of Medicine, Durham, NC, United States; ^2^Convergent Engineering Inc., Gainesville, FL, United States

**Keywords:** mechanical ventilation, ventilator sharing, ventilator splitting, remote ventilator monitoring, COVID-19, mass casualty events, personalized ventilator sharing, ventilator shortage

## Abstract

**Background:**

During the COVID-19 pandemic surge in the hospitalization of critically ill patients and the global demand for mechanical ventilators, alternative strategies for device sharing were explored. We developed and assessed the performance of a system for shared ventilation that uses clinically available components to individualize tidal volumes under a variety of clinically relevant conditions. The feasibility of remote monitoring of ventilators was also assessed.

**Methods:**

By using existing resources and off-the-shelf components, a ventilator-sharing system (VSS) that ventilates 2 patients simultaneously with a single device, and a ventilator monitoring system (VMS) that remotely monitors pulmonary mechanics were developed. The feasibility and effectiveness of VSS and VMS were evaluated in benchtop testing using 2 test lungs on a single ventilator, and then performance was assessed in translational swine models of normal and impaired lung function.

**Results:**

In benchtop testing, VSS and VMS delivered the set individualized parameters with minimal % errors in test lungs under pressure- and volume-regulated ventilation modes, suggesting the highest precision and accuracy. In animal studies, the VSS and VMS successfully delivered the individualized mechanical ventilation parameters within clinically acceptable limits. Further, we found no statistically significant difference between the target and measured values.

**Conclusion:**

The VSS adequately ventilated 2 test lungs or animals with variable lung conditions. The VMS accurately displayed mechanical ventilation settings, parameters, and alarms. Both of these systems could be rapidly assembled for scaling up to ventilate several critically ill patients in a pandemic or mass casualty disaster situations by leveraging off-the-shelf and custom 3D printed components.

## Introduction

During the initial surge of the COVID-19 (coronavirus disease 2019, SARS-CoV-2) pandemic, there was a potential global shortage of mechanical ventilators. Due to the severity of the COVID-19 sickness in patients, the need for mechanical ventilation was the most important predictor of mortality as it was associated with an approximately 200-fold increase in odds of death (1). The potential burden of mechanical ventilator shortage could be catastrophic, required stakeholders to evaluate capabilities, and triggered a rapid production of thousands of devices (2). In the early phases of COVID-19, many prototype ventilators were proposed, and a few locations used a single device to ventilate multiple patients (3–5). Respiratory and intensive care associations published information highlighting the potential dangers of using one device to simultaneously deliver mechanical ventilation to multiple patients (6, 7). There are several safety, physiological, and ethical concerns regarding ventilator sharing including: (1) tidal volumes (V_t_) would go to the most compliant lung segments; (2) positive end-expiratory pressure (PEEP) would not be individually managed; (3) monitoring patients and measuring pulmonary mechanics would be challenging; (4) alarm monitoring and management would not be feasible; (5) individualized management for clinical improvement would be impossible, and (6) if manual bag ventilation is required, it would need to occur without exposing clinicians to airborne pathogens and the second patient would be at risk; (7) Without proper filters or one-way valves as safeguards, there is a signification risk of cross-contamination between patients sharing the same ventilator circuit. Ventilator sharing would alter breath delivery dynamics to other patients, as the added circuit volume would defeat the operational self-test, i.e., the test fails. Even if all patients connected to a single ventilator had the same clinical features at initiation, they could deteriorate and recover at different rates, and the distribution of gas to each patient would be neither equal nor monitored. The sickest patient may receive the smallest V_t_, and the improving patient would get the largest V_t_; the greatest risks would occur if a single patient suddenly deteriorated, e.g., pneumothorax or kinked endotracheal tube, and the balance of ventilation was distributed to the other patients. Finally, there are ethical issues. If the ventilator could be lifesaving for a single individual, its use on more than one patient at a time would risk life-threatening treatment failure for all of them (8).

Despite these concerns, a ventilator sharing approach could be beneficial during the pandemic or in possible future mass casualty situations. Limited preclinical and clinical studies have shown promising results (3, 6, 9–13). However, limitations of previously published prototypes of ventilator sharing approaches may have hindered the technological development. During the COVID-19 pandemic, the shortage of supplies and equipment, and the strain on clinical personnel were a global concern (1, 2). A potential solution could be remote monitoring of patients on a mechanical ventilator to improve efficiency and minimize the use of items in short supply, such as personal protective equipment (PPE). To address the limitations of existing prototypes of ventilator sharing approaches and a need for remote monitoring, 2-part ventilator-sharing system (VSS) (Vent Guard, Convergent Engineering Inc., Gainesville, FL, United States) and ventilator monitoring system (VMS) were developed (14–16). The purpose of this study was to evaluate the precision and accuracy of mechanical ventilation monitoring and sharing, initially on a benchtop system using two test lungs and then in a swine translational model. Our overall goal was to validate the function of the device and to test the feasibility of translation to human clinical care applications.

## Materials and methods

### System design

The VentGuard system consists of two parts – VMS and VSS. The VMS uses a 3D-printed electronic pressure-flow sensor, and the user interface was built on an Android platform, with software developed to monitor respiratory parameters (Convergent Engineering). The VSS builds upon the VMS by adding a custom 3D-printed pneumatic valve similar to a PEEP valve, to control the flow of gas to a patient and software on the VMS to control the valve (Supplementary Figures S1, S2). For more details, *see the* *Supplementary material*. The VSS included an additional control panel that sets the target V_t_ or inspiratory pressure (IP), individual controls, alarms, and remote monitoring. Supplementary Figure S3 shows representative waveforms, numeric data of respiratory parameters, and alarm setting features displayed on the VMS and VSS. The VMS system also provides remote monitoring for any ventilator that displays alarms and respiratory data outside of a patient care room (Supplementary Figure S4). This allows clinicians to monitor the patient without having to enter the isolation room and to conserve resources and time to don PPE materials.

### Benchtop tests

**Figure 1:**
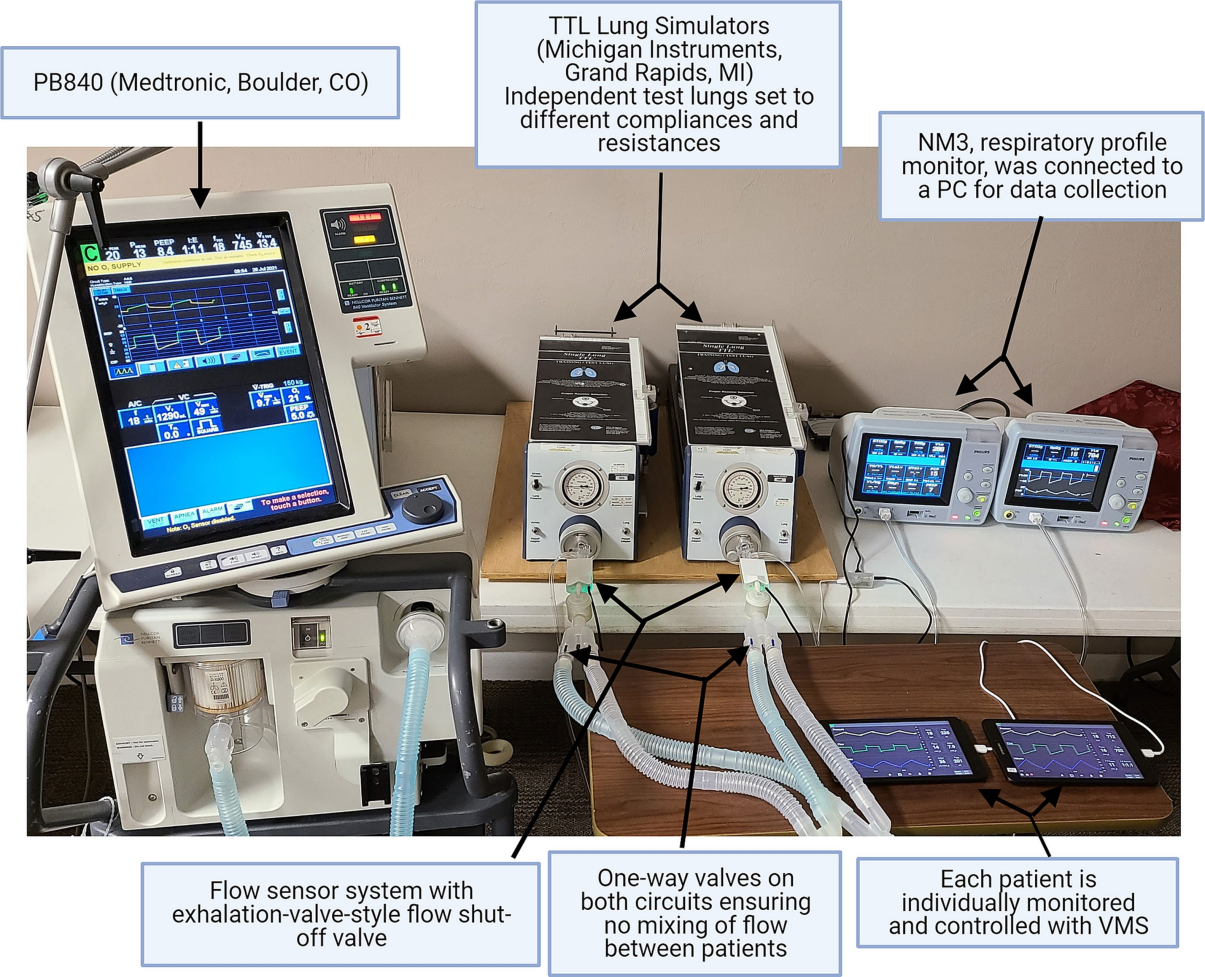
Benchtop test set-up. In benchtop testing, a single ventilator (PB840, Medtronic, Boulder, CO, United States) was connected to two test lungs. The performance of the ventilator monitoring system (VMS) was evaluated and compared using the standard NM3 monitor.

Bench tests were conducted to validate the system. A mechanical ventilator (Puritan Bennet 840, Medtronic, Boulder, CO, United States) was used to ventilate two different test lungs simultaneously (Michigan Instruments, Model 4600 Single Lung TTL^®^, Grand Rapids, MI, United States). The ventilator circuit was split using Y-adapters on both the inhaled and exhaled limbs of the circuit. Both test lungs were equipped with one-way valves for inhalation and exhalation placed before the circuit Y-piece to prevent flow (and potentially viruses/bacteria during clinical use) from moving between patients. The circuit Y-piece was connected to the VSS valve and sensor on each test lung, and also to a respiratory monitor (NM3, Phillips HealthCare, Carlsbad, CA, United States), which was used to validate the performance of the systems (Figure 1; Supplementary Figure S5).

A combination of settings was used for benchtop testing. For pressure-controlled ventilation (PCV) mode, we set IP values of 8, 12, and 16 cmH_2_O with test lung compliance (Crs) values of 25 and 50 mL/cmH_2_O and resistance (R) values of 5 and 20 cmH_2_O/L/s. Similarly, for volume-controlled (VC) mode, V_t_ was set at 300, 500, and 720 mL with Crs values of 25 and 50 mL/cmH_2_O and R values of 5 and 20 cmH_2_O/L/s.

### *In vivo* preclinical studies

Studies with animal models were conducted to validate the application and accuracy of the VSS. The goals for preclinical testing were: (1) to evaluate the VMS by comparing it with an NM3 respiratory profile monitor (Philips Healthcare, Wallingford, CT, United States), and (2) to evaluate the VSS by ensuring that it safely and reliably delivered and maintained the desired settings for individually ventilating two animals using one ventilator.

All procedures were approved by the Duke University Institutional Animal Care and Use Committee and were performed in accordance with the Guide for the Care and Use of Laboratory Animals. Yorkshire domestic swine (*n* = 5, male, 51–53 kg) were prepared for the studies as described previously (17, 18). Swine were fasted overnight before the studies; and premedicated with intramuscular (i.m) ketamine (22 mg/kg) and acepromazine (1.1 mg/kg). Buprenorphine (0.01 mg/kg, i.m) and carprofen (4 mg/kg, s.q) were administered as pre-emptive analgesia. Anesthesia was induced and maintained by propofol (4–6 mg/kg, IV). Animals were intubated with a 7.5 mm endotracheal tube, and connected to a mechanical ventilator (Avea, Vyaire, Riverside, CA, United States). Mechanical ventilation was initiated using a V_t_ of 7 mL/kg, a respiratory rate (RR) of 25 breaths per minute (bpm), PEEP of 5 cmH_2_O, and a fraction of inhaled oxygen (FiO_2_) of 21%. The neuromuscular blocker vecuronium bromide was administered (0.4 mg/kg IV bolus, and then maintained with 0.2–0.3 mg/kg/h IV) before initiating a warm saline lavage to induce mild acute respiratory distress syndrome (ARDS)-like phenotype, with some modifications of previously published approaches (19, 20). Briefly, the ventilator circuit was disconnected from the endotracheal tube. Pre-warmed sterile saline (1 liter bag at 37°C) was connected to a custom-made tubing that was attached to an endotracheal tube and elevated to let the saline flow into the lungs. Then the bag was lowered to allow broncho-alveolar lavage to flow out by gravity. The procedure was repeated with fresh warm saline with less than 5 min gap between each lavage procedure until SpO_2_ levels dropped below 90%.

**Figure 2:**
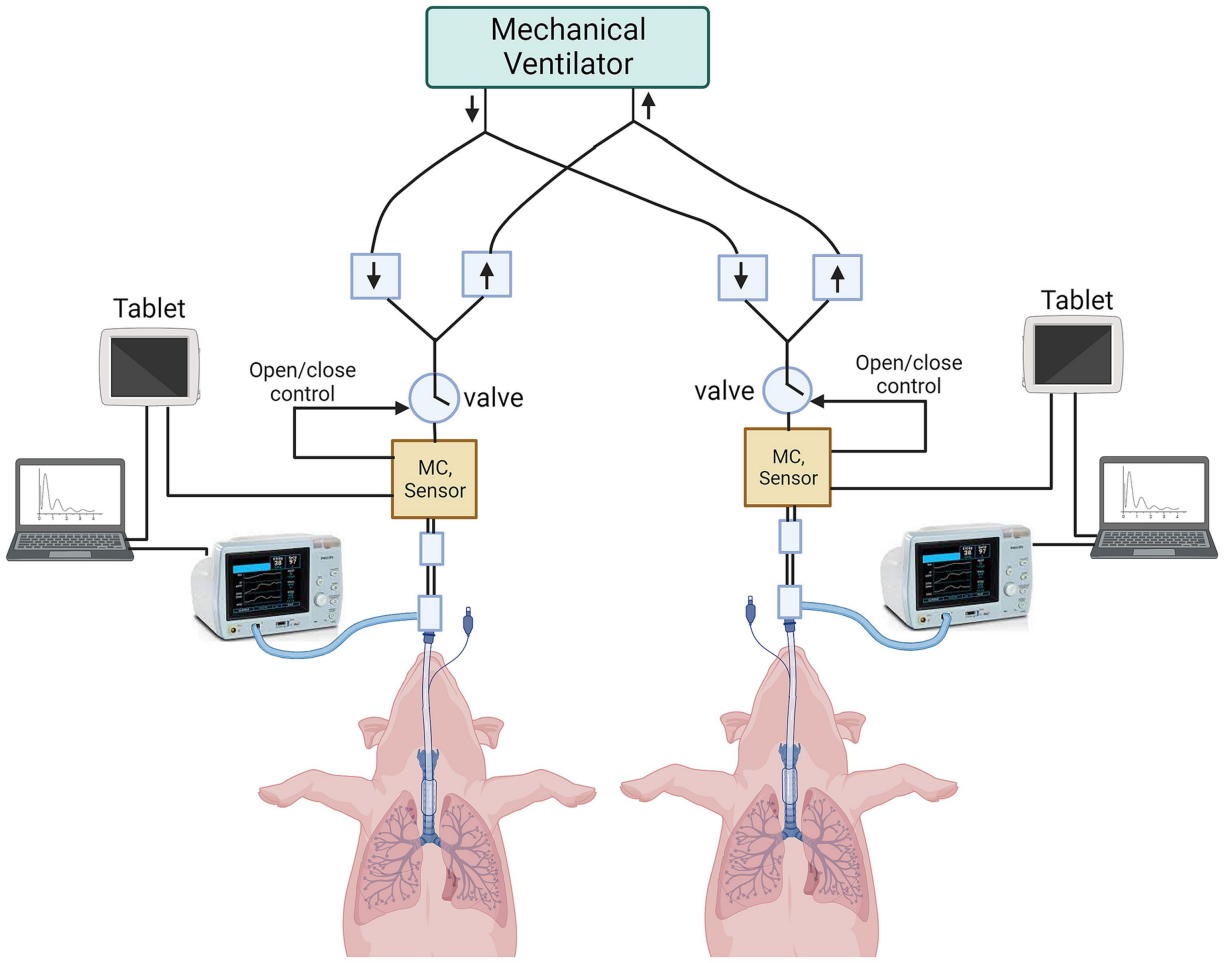
Schematic of preclinical animal testing in swine. Two anesthetized and orotracheally intubated pigs are connected to the ventilator splitter using VSS. The data are captured from VSS onto VMS tablets, and from NM3 profile monitors onto laptops. (MC = micro-controller).

A single mechanical ventilator was used to ventilate both animals using the VSS. Each animal was monitored by an NM3 respiratory profile monitor and VentGuard tablets. Laptop PCs were used to collect all streaming data (flow, pressure, volume, SpO_2_, PetCO_2_) and breath-based parameter data from both animals in real time using a proprietary software program (Venti, Convergent Engineering Inc). Similarly, the VentGuard system recorded streaming and breath-based data simultaneously directly on the tablets (Figure 2).

Three animal studies were conducted simulating different scenarios.

#### Study 1

The purpose of this *in vivo* study was to validate the VMS and VSS and corroborate with bench testing findings. A 51-kg pig was ventilated with and without spontaneous breathing using PCV and VC modes with multiple PEEP levels (5–10 cmH_2_O). Ventilation strategies were initially tested in healthy lungs and then subsequently in saline lavage-induced ARDS simulated conditions. Each setting was monitored and recorded for 10 min by both NM3 and VMS for analysis. To ensure the system would handle disturbances and erroneous data, several circuit disconnections and simulated coughs (forced expiratory oscillations) were used. In total, 10 different breath parameters across 19 different ventilator settings and scenarios were compared between VMS and NM3 under normal and impaired lung conditions.

#### Study 2

The purpose of this study was to evaluate the VMS and VSS in both healthy and impaired lungs over time. The VSS was evaluated by ventilating two animals simultaneously using one ventilator. Since a single ventilator cannot respond to patient effort simultaneously from two or more patients, only controlled mechanical ventilation was used. Two pigs (both 53 kg) were connected via Y-connectors, one-way valves, and the VSS to one ventilator. First, both healthy animals were ventilated for 100 min, and then 74 min after ARDS was induced by saline lavage. In both sessions, the VSS was set up to ventilate the animals with different individual V_t_ and IP settings. Initial ventilator settings were PCV 20 cmH_2_O, respiratory rate (RR) 12 bpm, inspiratory time (T_i_) 1.5 s (s), PEEP 5 cmH_2_O, and FiO_2_ 40%. In study 2, a total of seven settings were tested while both animals were healthy, and another seven settings were tested after the saline lavage procedure to mimic ARDS.

#### Study 3

The purpose of this study was to evaluate the VMS and VSS simulating two different patient conditions that changed over time. Lung injury was induced by repeated warm saline lavage in one pig (55 kg) while the other pig (52 kg) had healthy lungs, and data were recorded in six different settings. In the next scenario, ARDS was induced in the second animal while the first pig recovered slowly, thus providing a scenario in which each pig was in various states of health during the study. Study 3 also included 6 different settings.

At the end of each study, animals were euthanized following American Veterinary Medical Association (AVMA) guidelines. All data were analyzed using GraphPad Prism 10.1.2 for Windows (GraphPad Software, San Diego, CA, United States) or MATLAB (R2018a, MathWorks, Natick, MA, United States). Mean values between 2 different groups were compared by a 2-tailed Student’s *t-*test. Violin plots show the frequency distribution of the data along with the comparison of means of two data groups in comparison. Quantile-quantile (Q-Q) plots were presented to compare actual and predicted residuals. The distribution of residuals around the 45-degree reference line demonstrates the univariate normality of the data. Bland–Altman plots show the average bias (the average of the differences) of target and measured values or comparison of two systems. The average of the differences between the two systems will be close to zero if there is a good agreement between them. Statistical significance was denoted by *p < 0.05 or ns = non-significant (*p* > 0.05).

## Results

### Benchtop testing

The benchtop testing demonstrated high accuracy in delivering the set studied parameters. Tables 1, 2 show the results from benchtop testing in PCV and VC modes, respectively. We presented key parameters such as IP, PEEP, T_i_, RR, Crs, and R. In PCV mode, overall average percentage errors were 1.1 and 0.8 in the 2 test lungs. In VC mode, the average percent errors were 1.3 and 0.5. In both test modes, the mean difference between target pressure or volume and measured pressure or volume in test lung 1 or test lung 2 was not significantly different (2-tailed Student’s *t-*test, *p* > 0.05). Supplementary Figures S6, S7 show violin plots, residual plots, quantile-quantile (Q-Q) plots, and Bland–Altman plots for both tested ventilation modes. The mean target IP and V_t_ were not significantly different from the measured values in either test lung (2-tailed Student’s *t*-test, *p* > 0.05). Residual and Q-Q plots showed that the residuals are uniformly distributed. Bland–Altman plots showed minimal average discrepancy (the bias) of the target and measured values of the studied parameters.

**Table 1:** Evaluation of ventilator-sharing system (VSS) in benchtop testing using two test lungs under pressure control ventilation mode.

				Simulated patient 1					Simulated patient 2					Simulated patient 1		Simulated patient 2	
PB-840 PCV settings				Test lung		VSS setting	NM3 measurements		Test lung		VSS setting	NM3 measurements		Error IP	%Error IP	Error IP	%Error IP
IP	PEEP	T_i_	RR	Crs	R	IP	PEEP	PIP	Crs	R	IP	PEEP	PIP				
22	10	1.25	24	25	5	16	9.3	25.5	25	5	12	10.1	22.3	−0.2	1.3%	−0.2	0.8%
22	10	1.25	24	25	5	16	10.1	26.1	50	20	8	13	21.02	0	0.0%	−0.02	0.1%
22	10	1.25	24	25	20	16	9.7	25.4	25	20	12	10	21.8	0.3	1.9%	0.2	0.8%
22	10	1.25	24	25	20	16	10.4	26.5	50	5	8	11.8	20.1	−0.1	0.6%	−0.3	1.1%
26	15	1.25	24	50	5	16	15	31.1	50	5	8	14.3	22.2	−0.1	0.6%	0.1	0.3%
26	15	1.25	24	50	5	16	14.4	30.6	25	20	12	12.1	23.9	−0.2	1.3%	0.2	0.7%
28	15	1.25	24	50	20	16	17	33.4	50	20	8	13.8	22.1	−0.4	2.5%	−0.3	0.9%
28	15	1.25	24	50	20	16	16.3	32.5	25	5	12	11.3	23	−0.2	1.3%	0.3	0.9%
22	10	1.25	12	25	5	16	9.6	25.8	25	5	12	10.1	21.9	−0.2	1.3%	0.2	0.8%
22	10	1.25	12	25	5	16	9.6	25.6	50	20	8	10.1	18.2	0	0.0%	−0.1	0.4%
22	10	1.25	12	25	20	16	9.7	25.7	25	20	12	10.2	22.4	0	0.0%	−0.2	0.8%
22	10	1.25	12	25	20	16	9.6	26	50	5	8	10.1	17.9	−0.4	2.5%	0.2	0.8%
22	10	1.25	12	50	5	16	9.6	25.5	50	5	8	10.1	18.3	0.1	0.6%	−0.2	0.8%
22	10	1.25	12	50	5	16	9.6	25.5	25	20	12	10.1	21.6	0.1	0.6%	0.5	2.0%
26	10	1.25	12	50	20	16	9.6	25.6	50	20	8	10.1	18	0	0.0%	0.1	0.4%
26	10	1.25	12	50	20	16	9.5	25.1	25	5	12	10.1	22.3	0.4	2.5%	−0.2	0.8%
													Mean	−0.06		0.02	
													Stdev	0.22		0.24	
													Absolute mean % error		1.1%		0.8%

PCV = pressure control ventilation mode; VSS = ventilator-sharing system; IP = inspiratory pressure (cmH_2_O); PEEP = positive end expiratory pressure (cmH_2_O); PIP = peak inspiratory pressure (cmH_2_O); T_i_ = inspiratory time (s); RR = respiratory rate (breaths per minute, bpm); Crs = dynamic compliance (mL/cmH_2_O); R = resistance (cmH_2_O/L/min); stdev = standard deviation.

**Table 2:** Evaluation of ventilator-sharing system (VSS) in benchtop testing using two test lungs under volume control mode.

				Simulated patient 1					Simulated patient 2					Simulated patient 1		Simulated patient 2	
PB-840 VC settings				Test lung		VSS setting	NM3 measurements		Test lung		VSS setting	NM3 measurements		Error V_t_	%Error V_t_	Error V_t_	%Error V_t_
IP	PEEP	T_i_	RR	Crs	R	V_t_	PEEP	V_t_	Crs	R	V_t_	PEEP	V_t_				
22	10	1.25	24	25	5	300	9.4	297	25	5	500	9.8	503	−3	1.0%	3	0.6%
22	10	1.25	24	25	5	300	10.8	303	50	20	720	16	718	3	1.0%	−2	0.3%
22	10	1.25	24	25	20	300	10	302	25	20	500	10.8	500	2	0.7%	0	0.0%
22	10	1.25	24	25	20	300	10.6	296	50	5	720	13.7	727	−4	1.3%	7	1.0%
22	10	1.25	24	50	5	300	12.4	308	50	5	720	13.6	721	8	2.7%	1	0.1%
22	10	1.25	24	50	5	300	11	293	25	20	500	10.7	502	−7	2.3%	2	0.4%
26	10	1.25	24	50	20	300	12.5	291	50	20	720	16.2	712	−9	3.0%	−8	1.1%
26	10	1.25	24	50	20	300	11.8	302	25	5	500	10.4	501	2	0.7%	1	0.2%
22	10	1.25	12	25	5	300	9.7	298	25	5	500	10.1	498	−2	0.7%	−2	0.4%
22	10	1.25	12	25	5	300	9.6	308	50	20	720	10.1	722	8	2.7%	2	0.3%
22	10	1.25	12	25	20	300	9.7	299	25	20	500	10.1	500	−1	0.3%	0	0.0%
22	10	1.25	12	25	20	300	9.8	296	50	5	720	10.3	728	−4	1.3%	8	1.1%
22	10	1.25	12	50	5	300	9.7	303	50	5	720	10.1	715	3	1.0%	−5	0.7%
22	10	1.25	12	50	5	300	9.6	301	25	20	500	10.1	498	1	0.3%	−2	0.4%
22	10	1.25	12	50	20	300	9.6	298	50	20	720	10.1	718	−2	0.7%	−2	0.3%
22	10	1.25	12	50	20	300	9.8	301	25	5	500	10.2	498	1	0.3%	−2	0.4%
												Mean		−0.25		0.06	
												Stdev		4.73		4.01	
												Absolute mean % error			1.3%		0.5%

VC = volume control ventilation mode; IP = inspiratory pressure (cmH_2_O); PEEP = positive end expiratory pressure (cmH_2_O); T_i_ = inspiratory time (s); RR = respiratory rate (breaths per minute, bpm); Crs = dynamic compliance (mL/cmH_2_O); R = resistance (cmH_2_O/L/s); V_t_ = tidal volume (mL); stdev = standard deviation.

### *In vivo* preclinical testing

The VSS functioned as designed. All animal studies were performed without any technical difficulties. The mechanical ventilation and circuit connections did not create any unforeseen events. In the first animal study, the studied variables [inspired tidal volume (VTi), expired tidal volume (VTe), average tidal volume (VTave), PEEP, mean airway pressure (mPaw), peak inspiratory flow (PIF), peak expiratory flow (PEF), RR, Ti, and expiratory time (Te)] maintained high fidelity between VMS and NM3 recordings. The data from study 1 (Supplementary Table S1) shows that the performance of the VMS was confirmed with an NM3 respiratory profile monitor in a pig under normal and impaired lung conditions. The presented *p*-values for each parameter and each setting indicate that if the *p*-value is <0.05, the means are very likely to be within 10% of each other, but if the *p*-value >0.05, it is not statistically proven that they are within 10% of each other.

**Figure 3:**
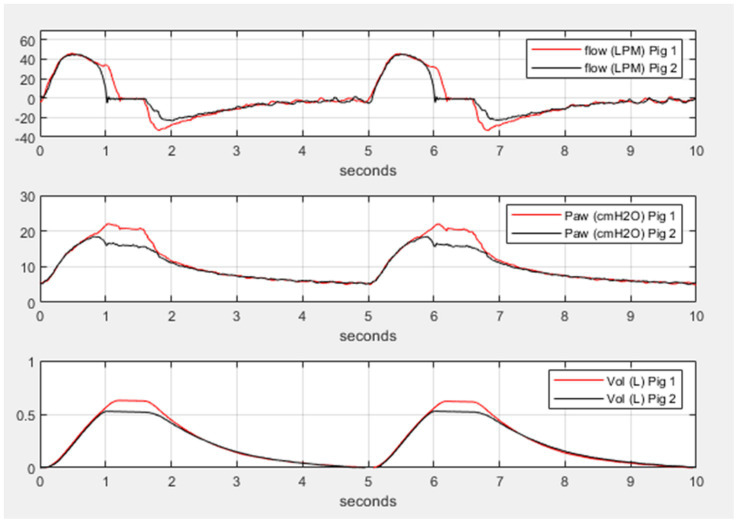
Differential and personalized mechanical ventilation in two pigs. Representative simultaneous breaths from two pigs using the ventilator-sharing system (VSS). In all three panels, pig 1 has a target pressure of 20 cmH_2_O and pig 2 has a target pressure of 15 cmH_2_O. The top panel shows the flow for each pig, the middle panel shows the airway pressure for each pig, and the bottom panel shows the volume for each pig. Inhalation time in the pressure control ventilation was set to 1.5 s but pig 1 received longer inhalation flow and higher volume due to the higher target pressure illustrating the individual control of pressure and tidal volume.

**Figure 4:**
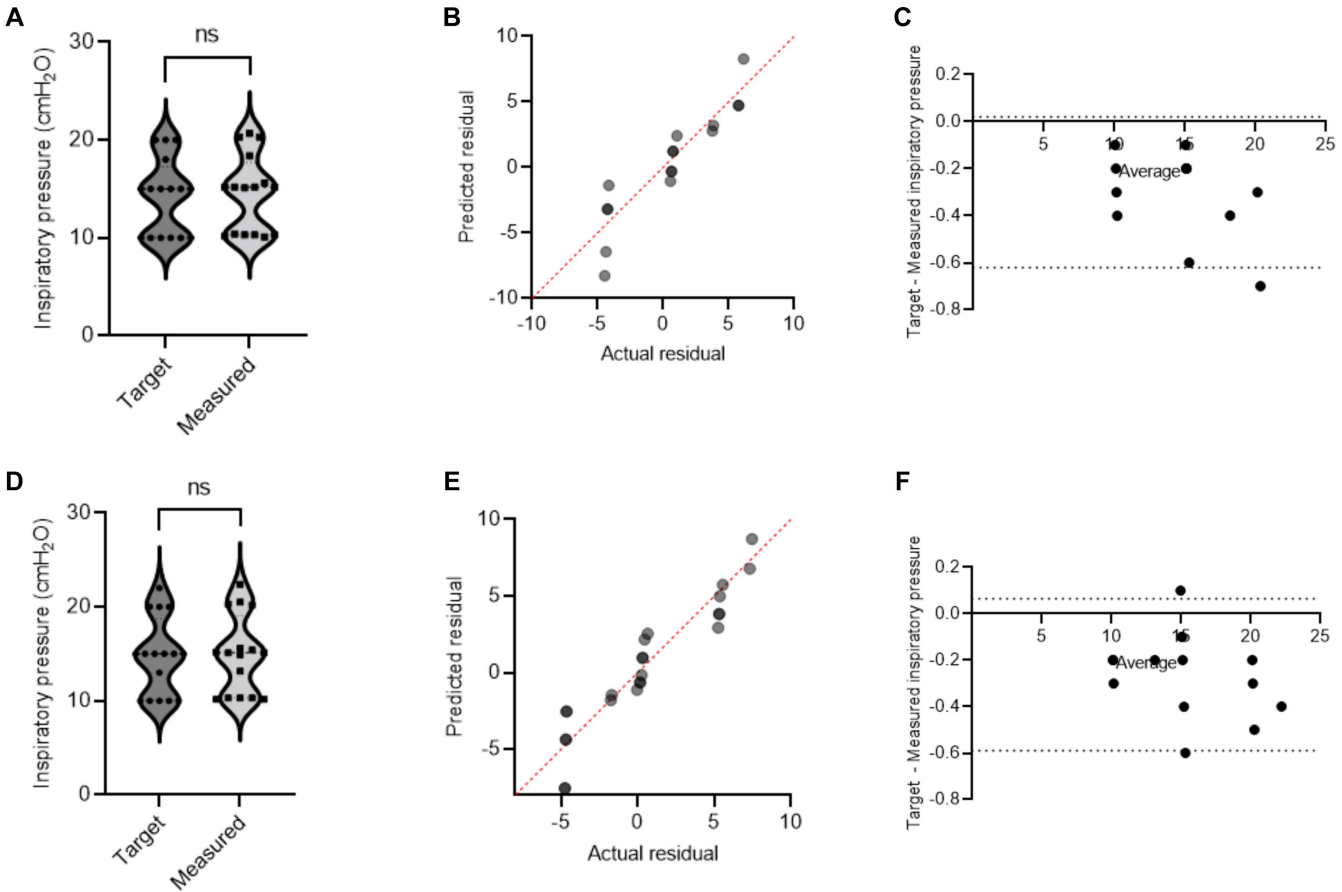
A single ventilator delivers set inspiratory pressure to two pigs. In swine preclinical studies, a single ventilator was used to deliver a set inspiratory pressure (IP) to two swine at a time using VSS. The data were collected from swine under normoxia and hypoxia [acute respiratory distress syndrome (ARDS)-like injury phenotype caused by warm saline lavage]. **(A–C)** and **(D–F)** show violin plots, quantile-quantile (Q-Q) plots, and Bland–Altman plots. The data are presented from four swine.

**Figure 5:**
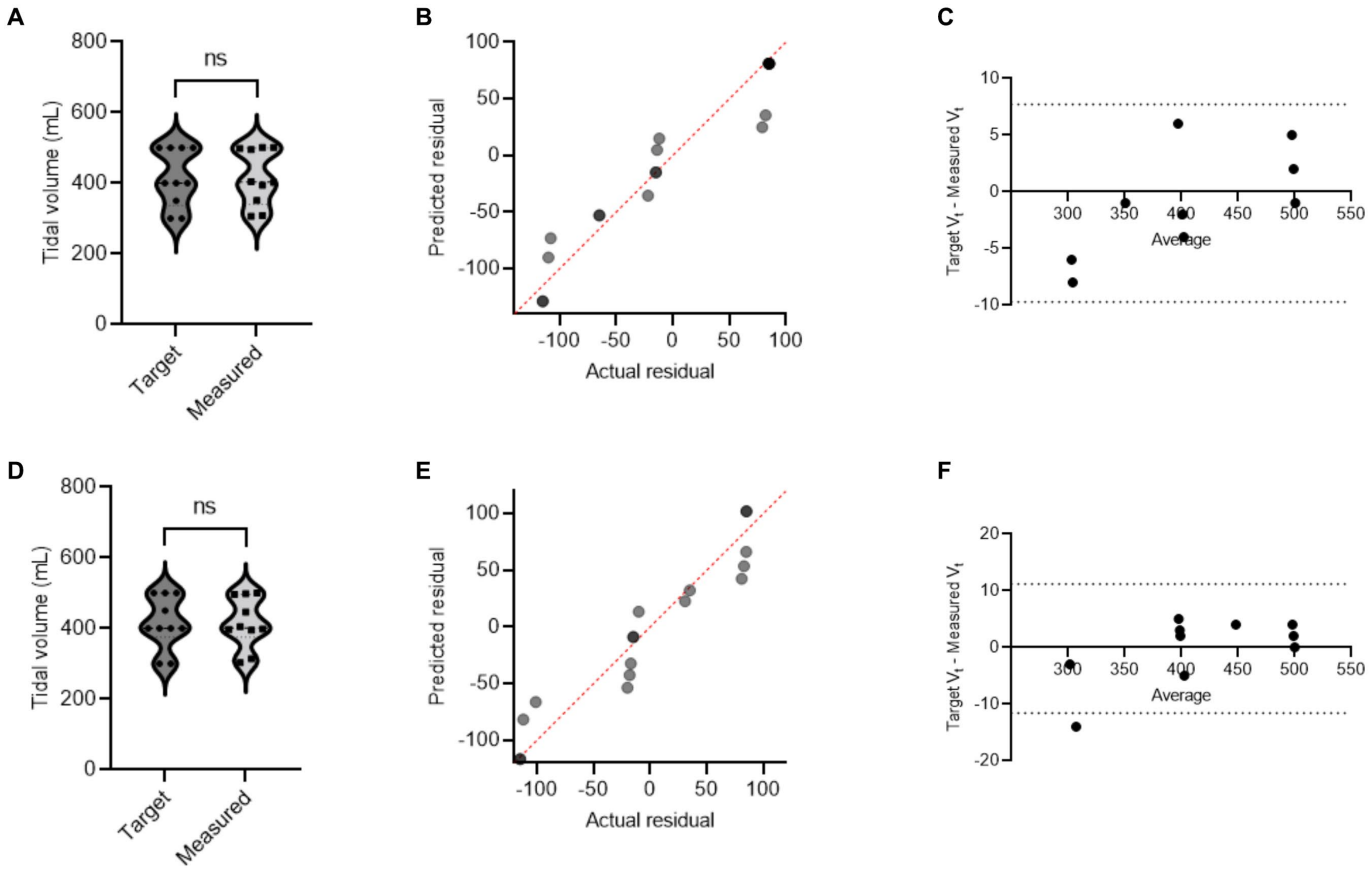
A single ventilator delivers set tidal volume to two swine. In swine preclinical studies, a single ventilator was used to deliver a set tidal volume (V_t_) to two swine at a time using a ventilator-sharing system (VSS). The data were collected from swine under normoxia and hypoxia [acute respiratory distress syndrome (ARDS)-like phenotype caused by warm saline lavage]. **(A–C)** and **(D–F)** show violin plots, quantile-quantile (Q-Q) plots, and Bland–Altman plots. The data are presented from four swine.

During mechanical ventilation sharing in two pigs, when one animal reached its respective target volume or pressure before the other, that animal would stop receiving flow and have a longer end-inspiratory pause (EIP). Figure 3 shows a schematic demonstration of personalized control of ventilation for two animals with different ventilation needs. The data from Study 2 and Study 3 were combined, and data for target and actual measured pressure and volume were presented under different health conditions (Supplementary Table S2). In our mild ARDS model, although SpO_2_ values dropped below 90% at the end of the lavage procedure, animals recovered from hypoxia rapidly. Figures 4, 5 show violin plots, quantile-quantile (Q-Q) plots, and Bland–Altman plots comparing individual target inspiratory pressure or V_t_ with respective measured values in simultaneously ventilated animals. In Bland–Altman plots, the bias (mean difference) for the inspiratory pressure study parameter for pig 1 and pig 2 was −0.3 and −0.26, respectively; whereas the bias for V_t_ for pig 1 and pig 2 was −1 and −2, respectively. The targets for inspiratory pressure and V_t_ were not significantly different from the measured values in both pigs (2-tailed Student’s *t-*test, *p* > 0.05).

## Discussion

We developed a novel ventilator-splitting device in response to the COVID-19 pandemic and possible future pandemic or mass casualty situations in which the number of patients exceeds the available ventilators. A new remote monitoring system was also developed and tested to simulate patient care scenarios. Our primary findings showed that a system can be developed and used to provide adequate ventilatory support and remote monitoring to multiple patients with variable lung conditions using a single ventilator. The VSS addresses most of the limitations of previous studies, with the exceptions of individual control of PEEP, the inability to accurately conduct self-tests on a shared ventilator, and spontaneous breathing by multiple patients (5–7, 13, 21, 22).

Benchtop testing demonstrated high precision and accuracy in delivering mechanical ventilation in both PCV and VC modes. More interestingly, we chose the values for our benchtop testing based on the published respiratory physiologic values in large COVID-19 cohort studies (23–27). The high correlation of values recorded on VSS and NM3 suggests comparable performance relative to an FDA-approved clinical respiratory profile monitor. In benchtop testing, in pressure control mode under low compliance conditions, we could not perform testing at an inspiratory pressure of 8 cmH_2_O as it created a V_t_ that was too small. Therefore, under such scenarios, we used an IP value of 12 cmH_2_O. Similarly, for the low compliance VC mode, the highest V_t_ (720 mL) was not achieved safely; therefore, we reduced V_t_ to 500 mL when the compliance was very low.

*In vivo* testing to assess the functionality of VSS and VMS in comparison with NM3 respiratory profile monitor showed high agreement in their performance. The VSS provided individualized IP and V_t_ to two pigs simultaneously with different target values under varying health simulated conditions. Further, the VMS provides low-cost, easily manufactured remote respiratory monitoring for any ventilator system, and is particularly advantageous in situations when remote monitoring is beneficial or required. The system addresses several of the limitations that were not addressed in COVID-19 pre-pandemic attempts at ventilator sharing, such as individualized ventilation for each patient, dynamic rebalancing of the airflow (pressure or volume), and real-time measurement of pulmonary mechanics.

While the implementation of ventilator sharing is not optimal and should be used only in specific situations, preparation for such situations includes properly designed and tested systems (6, 12, 28). Systems must be easy to use, accurate, and focused on safety to prevent inadvertent harm to patients. Several studies have proposed and tested ventilator-sharing strategies, but most have been theoretical, computational, or bench-tested only (14, 15). However, a few cases of ventilator sharing have been tested in limited preclinical studies or reported in ICU patients for brief periods of time (16). In this study, we demonstrated ventilation sharing using two different kinds of ventilators in benchtop and animal model scenarios. Our preclinical studies not only compared the performance of VSS with NM3 but also evaluated the feasibility and application of VSS to ventilate two pigs under varying pulmonary health conditions. Our system was able to electronically control and adjust for V_t_ and IP in healthy and injured lungs simultaneously. The breadth of scenarios evaluated suggested a robust assessment of VSS and its performance. Similar to our work, in a recent study, a pressure-regulated mechanical ventilation sharing was demonstrated initially *in vitro* using elastomeric lungs and then in two sheep (29). Some of the ventilator-sharing approaches have also been tested in 7 patients. Out of 7, five patients were paired with a test lung and two patients were ventilated together (30). Both patients ventilated synchronously while maintaining ventilatory, hemodynamic, and oxygenation parameters similar to our study.

While we addressed several of the limitations noted in prior studies on mechanical ventilator sharing and multiplexing, our study has limitations. We did not test prospectively if our one-way valve can prevent transmission of viral and bacterial infections among the patients connected to the VSS. Future studies are warranted to test if the one-way valve in our system can prevent cross-contamination. We also did not evaluate the potential for additional ventilator-induced lung injury (VILI) from the splitting ventilator as this requires extended mechanical ventilation and observations or traumatic ventilation approaches. Additionally, to simulate pandemic situations, gas exchange was monitored via capnography and pulse oximetry rather than arterial blood gas analysis. Our proof-of-concept preclinical studies were carried out in 5 animals without power analysis, yet we showed data under different simulative conditions. Despite minor limitations, our incremental approach in benchtop testing and then *in vivo* preclinical testing in translational swine models showed positive outcomes paving the way for further evaluation of ventilator multiplexing in animal models for extended observation studies and eventual clinical trials.

### Clinical implications

Our system has one-way valves in the breathing circuit, pressure regulators at each inspiratory limb of the circuit, inspiratory and expiratory flow sensors, and pressure sensors to personalize mechanical ventilation needs for each individual patient connected to the multiplexed mechanical ventilation sharing system. Overall, our system complies with the guidelines from the United States Food and Drug Administration (US FDA) for the development and use of ventilator-sharing systems (28).

## Conclusion

Overall, we demonstrated a system that provides remote monitoring, and can facilitate personalized mechanical ventilation to two patients using a single device. While it is not ideal to promote or commonly use ventilator sharing in clinical settings, the COVID-19 pandemic forced many institutions to make unprecedented preparations and consider difficult decisions. The purpose of this project was to develop and test a system that could be used in such dire emergencies. Further, our monitoring system provides an accurate display of information and alarms outside of a patient’s room, which may help to provide efficient care, and reduce the need for unnecessary entry into a room during resource-limited care situations. Further research is warranted to evaluate any impact of mechanical ventilator sharing on clinically measured outcomes.

## Data availability statement

The original contributions presented in the study are included in the article/Supplementary material, further inquiries can be directed to the corresponding author.

## Ethics statement

The animal study was approved by Duke University Institutional Animal Care and Use Committee. The study was conducted in accordance with the local legislation and institutional requirements.

## Author contributions

SA: Conceptualization, Data curation, Formal analysis, Funding acquisition, Investigation, Methodology, Project administration, Resources, Supervision, Validation, Visualization, Writing – original draft, Writing – review & editing. MG: Conceptualization, Investigation, Methodology, Writing – original draft, Writing – review & editing. NRE: Conceptualization, Data curation, Formal analysis, Funding acquisition, Investigation, Methodology, Software, Visualization, Writing – original draft, Writing – review & editing.
