# Supplementary material for: Development and evaluation of a mechanical ventilator-sharing system
Source: Front Med (Lausanne). 2024 Feb 16;11:1356769. doi: 10.3389/fmed.2024.1356769 (PMC10905385; doi:10.3389/fmed.2024.1356769)
Supplement: Supplementary file 1 [file Data_Sheet_1.DOCX]

# **Development and Evaluation of a Mechanical Ventilator-sharing system**

# Satyanarayana Achanta, DVM, PhD, DABT^1*^; Michael A. Gentile, MBA, RRT, FAARC, FCCM^1^;

# Neil R. Euliano, PhD^2*^

# Affiliations:

# ^1^Department of Anesthesiology, Duke University School of Medicine, Durham, NC 27710

# ^2^Convergent Engineering, Gainesville, FL 32607

Keywords: mechanical ventilation, ventilator sharing, ventilator splitting, remote ventilator monitoring, COVID-19, mass casualty

*Corresponding Author:

Satya Achanta, DVM, PhD, DABT

Box 3094, MS27

Department of Anesthesiology

Duke University School of Medicine

Durham, NC 27710, USA

Email: satya.achanta@duke.edu

## **System Design**

The first part of the VentGuard system is a low-cost, easily manufactured, monitoring system based on a commercial respiratory monitor (RANDI) we are developing at Convergent Engineering. The system consists of a front-end transducer system derived from the RANDI monitor that is removably attached to a disposable airway sensor that can be 3D printed in emergencies or injection molded for mass production. The airway sensor is modeled after a standard Philips airway sensor and the front-end transducer system includes a low-cost microcontroller (MicroChip SAMD31) and the identical transducers used in RANDI. This transducer system communicates directly with a low-cost Android tablet (Samsung) that runs a modified version of the RANDI software. Each copy of the system was produced for approximately $250.

The VentGuard Monitoring System (VMS) shown in Figure S1 provides low-cost, easily manufactured, remote respiratory monitoring for any ventilator system, but is particularly advantageous for ventilators with minimal monitoring (such as emergency ventilators) or when remote monitoring is beneficial. The patient adapter includes a disposable airway adapter (D) that fits between the ventilator and the ET-Tube and contains two pressure taps around a low-pressure resistance to measure flow. Airway pressure is simultaneously measured from one of the two pressure taps on the patient adapter. The patient adapter was 3D printed and snaps into the VMS reusable electronic front end. The two pressure adapters plumbed directly to the pressure taps from the airway adapter are shown near label A. The differential pressure sensor is an AMS 6915-0010 (Analog Micro, Germany) with a range of +/- 10 mbar. The absolute pressure is measured with an AMS6915-0100 sensor with a range of 0 to 100 mbar. The small ARM microcontroller (C) (ATSAMD21G18A, MicroChip, Chandler, AZ) controls the front end, reads the pressures, and transmits/receives data from the user interface. A 3-way valve (X-Valve X-1-05-L-F, Parker Precision Fluidics, Hollis, HH) is included to charge and discharge the VentGuard Sharing System (VSS) flow control valve.

| 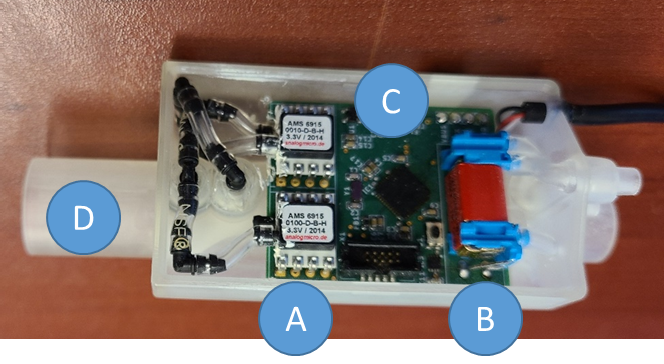 |
| --- |
| Figure S1. VentGuard Monitoring System. (A) Shows the differential (flow) and absolute (airway pressure) pressure sensors. (B) Shows the micro valve that turns on and off the VentGuard flow control valve (not used for monitoring only). (C) Shows the microcontroller. (D) shows the disposable airway adapter and flow sensor. The system is 1.5” x 2.75” x .75” high. |

The VentGuard Sharing System (VSS) includes the VMS electronic assembly and flow sensor (Figure S2, A & C) and a disposable pneumatic flow control valve (Figure 2, B) for controlling flow to the patient. The pneumatic flow control valve is a modified PEEP valve with a pneumatically powered diaphragm that controls flow through the circuit. The top of the diaphragm is loaded with pressure from the ventilator side of the circuit when the small electronic valve in the Monitoring System is turned on. Because the surface area at the top of the diaphragm is larger than the surface area of the blocked flow on the bottom, there is a mechanical advantage that allows the flow to the patient to be stopped using only airway pressure from the ventilator, without a separate high-pressure pneumatic source.

| 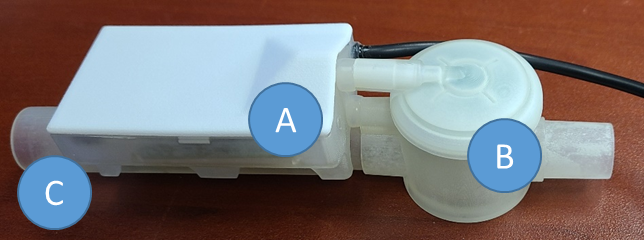 |
| --- |
| Figure S2. VentGuard Sharing System. (A) is the VMS electronics assembly, (B) is the VentGuard modified exhalation system for control of flow/pressure to the patient, (C) is the disposable VentGuard airway sensor for monitoring flow, volume, and pressure to the patient. The ET-Tube is connected at point (C) and the ventilator is connected at the inlet near point (B). The current system is powered and communicates via an extendable 4-wire connector. Adding wireless communication and a battery is feasible but introduces IT and recharging complexities. |

In the VSS, each patient has a VentGuard Sharing System front-end connected between the ventilator and the ET-Tube and a separate tablet for remote safety monitoring. The user interface on the tablet has settings for limiting either tidal volume (volume control) or plateau pressure (pressure control) for each individual patient. Because there are delays associated with the pneumatic flow control valve (Figure S2, B) that are pressure and flow-dependent, the system uses an adaptive system with extensive safety limits to quickly reach the desired volume or pressure levels for each patient.

The User Interface for the VentGuard system can be any commercial android system including mobile phones or tablets. In our studies, we used Samsung Tablets (Tab A, Samsung, Korea). The VentGuard software was modified from a commercial system Convergent Engineering is developing under an FDA-level Quality System. The user interface includes dynamic waveforms, important respiratory therapy parameters, alarms, and settings (Figure S3). The alarms are crucial to implementing a safe ventilator-sharing environment since the alarms on the shared ventilator will not work correctly when ventilating two patients.

| 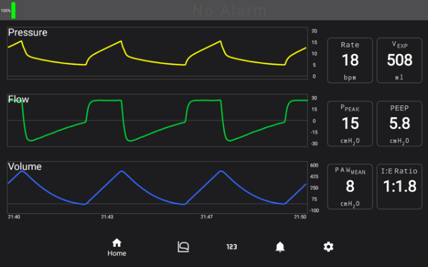 | 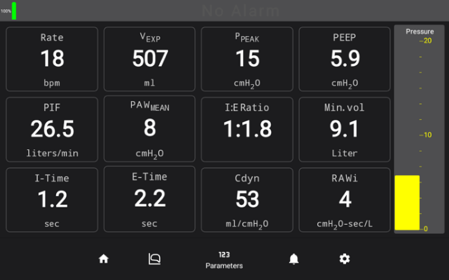 | 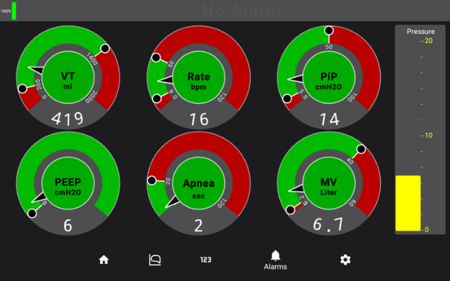 |
| --- | --- | --- |
| **Figure S3. Display of Ventilator-sharing system (VSS).** Left panel: real-time display of respiratory variables; Center panel: statistics of the most important respiratory variables; Right panel: Alarm selection | | |

The last component of the VentGuard system is a custom-designed splitter that allows the Android tablet to communicate with the microcontroller while enabling the charging and powering of both devices remotely. Several USB-splitting methods were attempted but none were reliable across all Android devices, thus the custom 4-wire interface to the microcontroller was used. The splitter is inexpensive, very small (1”x1”x.5”), and accepts power from an off-the-shelf USB power brick. It powers both systems and bridges the serial port data from the 4-wire microcontroller interface to the USB connector of the tablet using an FTDI FT312D IC.

Figure S4 shows an example of the VMS providing safety monitoring outside the patient room. This application allows alarms and respiratory data to be displayed outside the room (or applied to a window inside the room) to allow clinicians to safely monitor the patient without having to enter the isolation room. This application limits the time and PPE materials required for “gowning up” by the clinician on a daily basis.

| 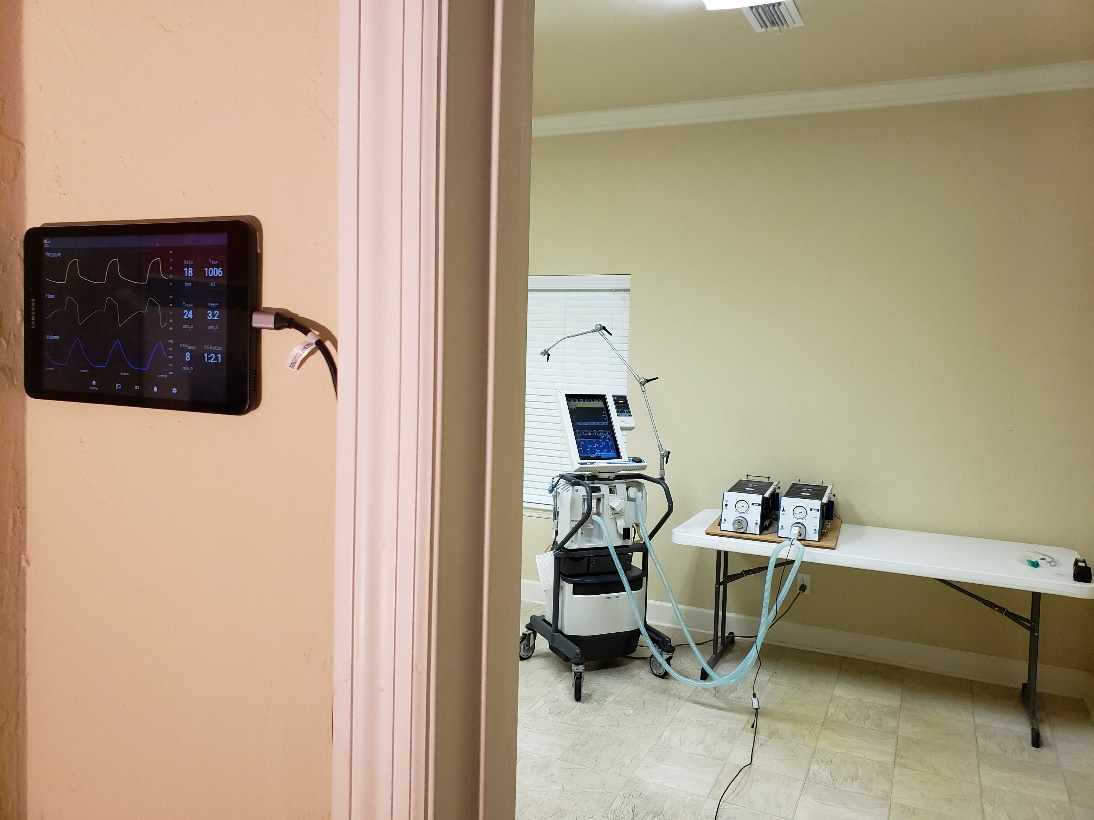 |
| --- |
| Figure S4. VentGuard monitoring system (VMS) demonstrating remote monitoring and alarms for use in isolation rooms. |


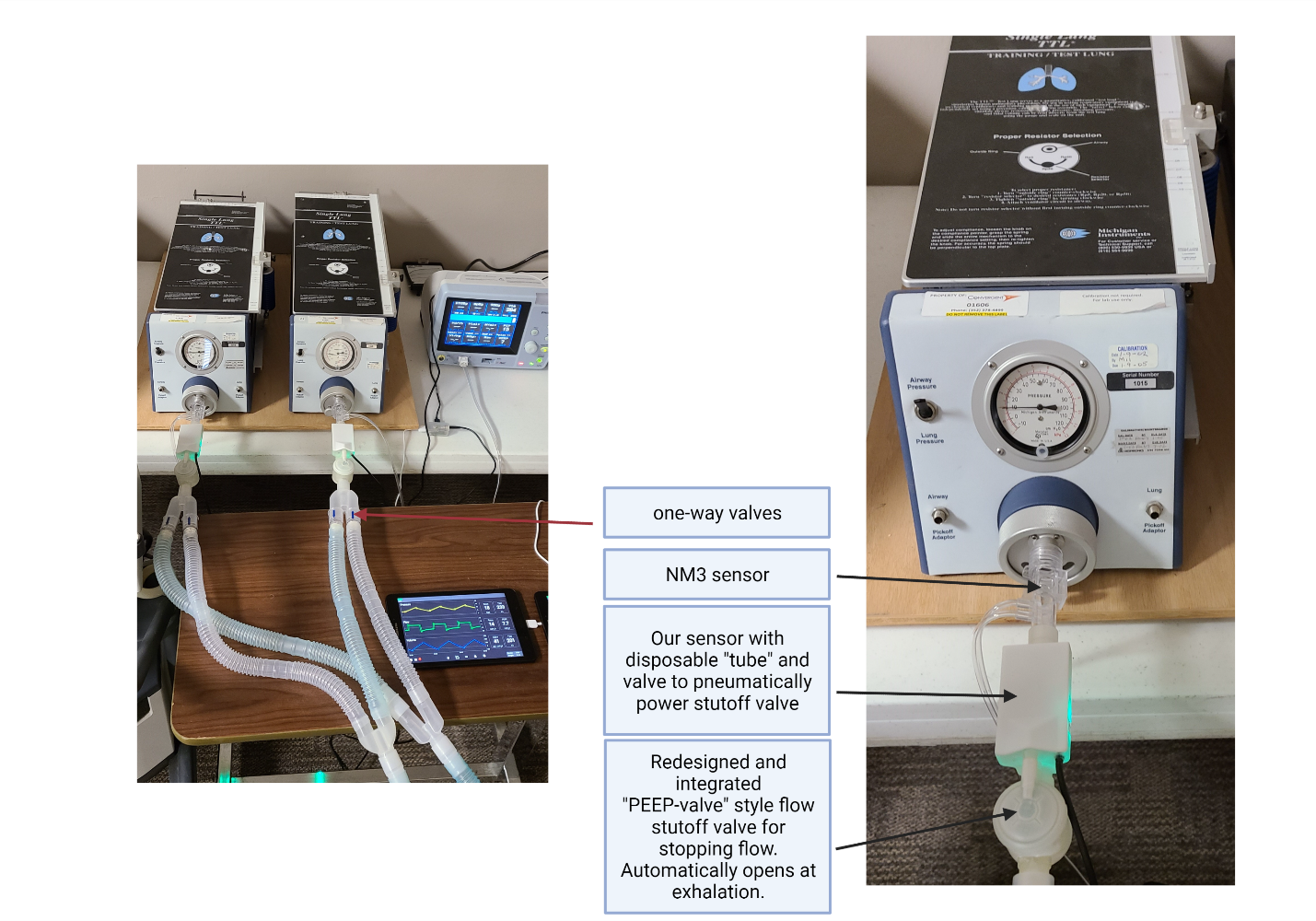


Figure S5: Magnified view of test lungs and circuit connections in benchtop testing set up.


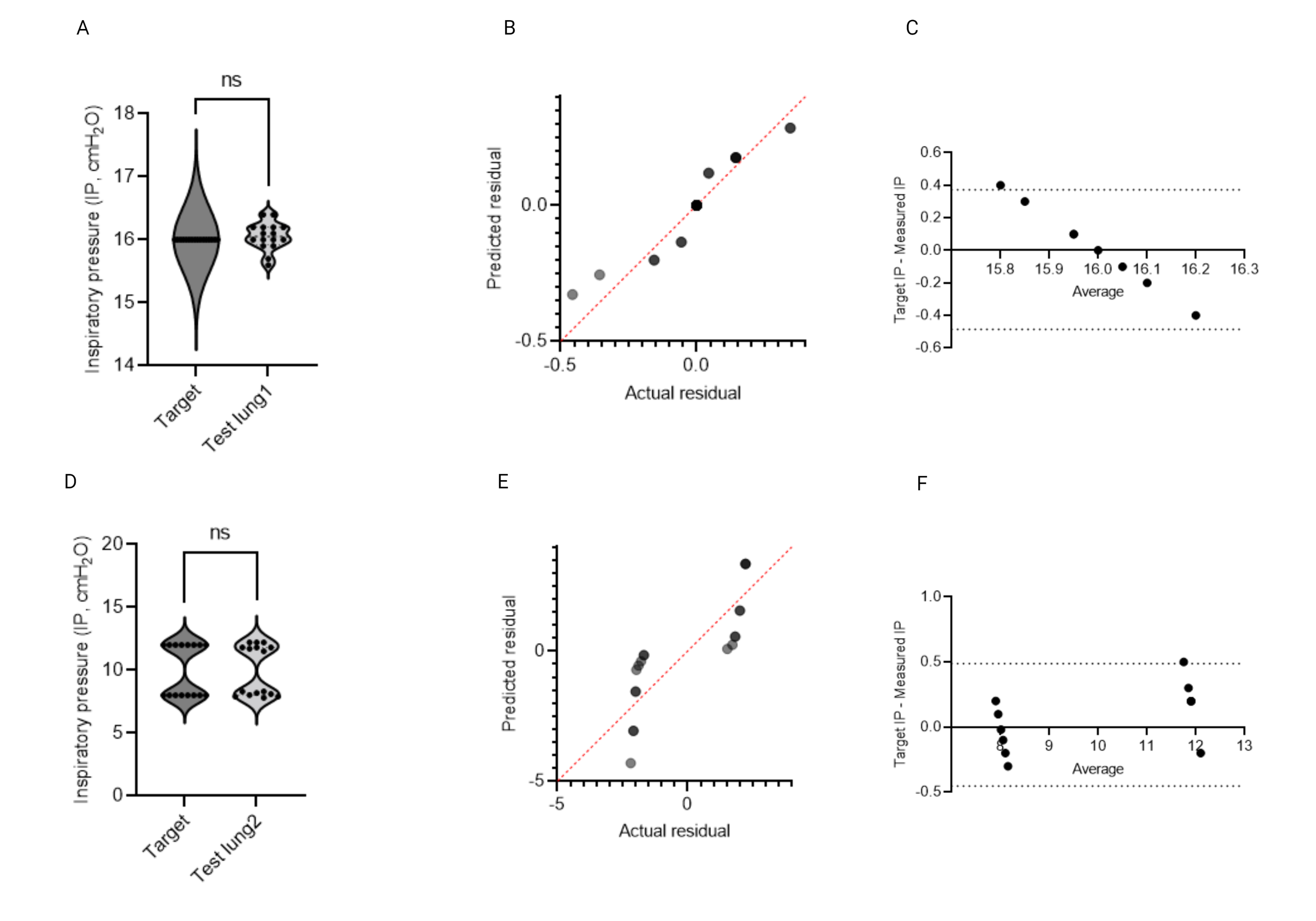


Figure S6. In benchtop testing, a single ventilator was employed to deliver set inspiratory pressure (IP) to two test lungs using VSS. Panels (A-C) and (D-F) show violin plots, quantile-quantile (Q-Q) plots, and Bland-Altman plots in test lungs 1 and 2.


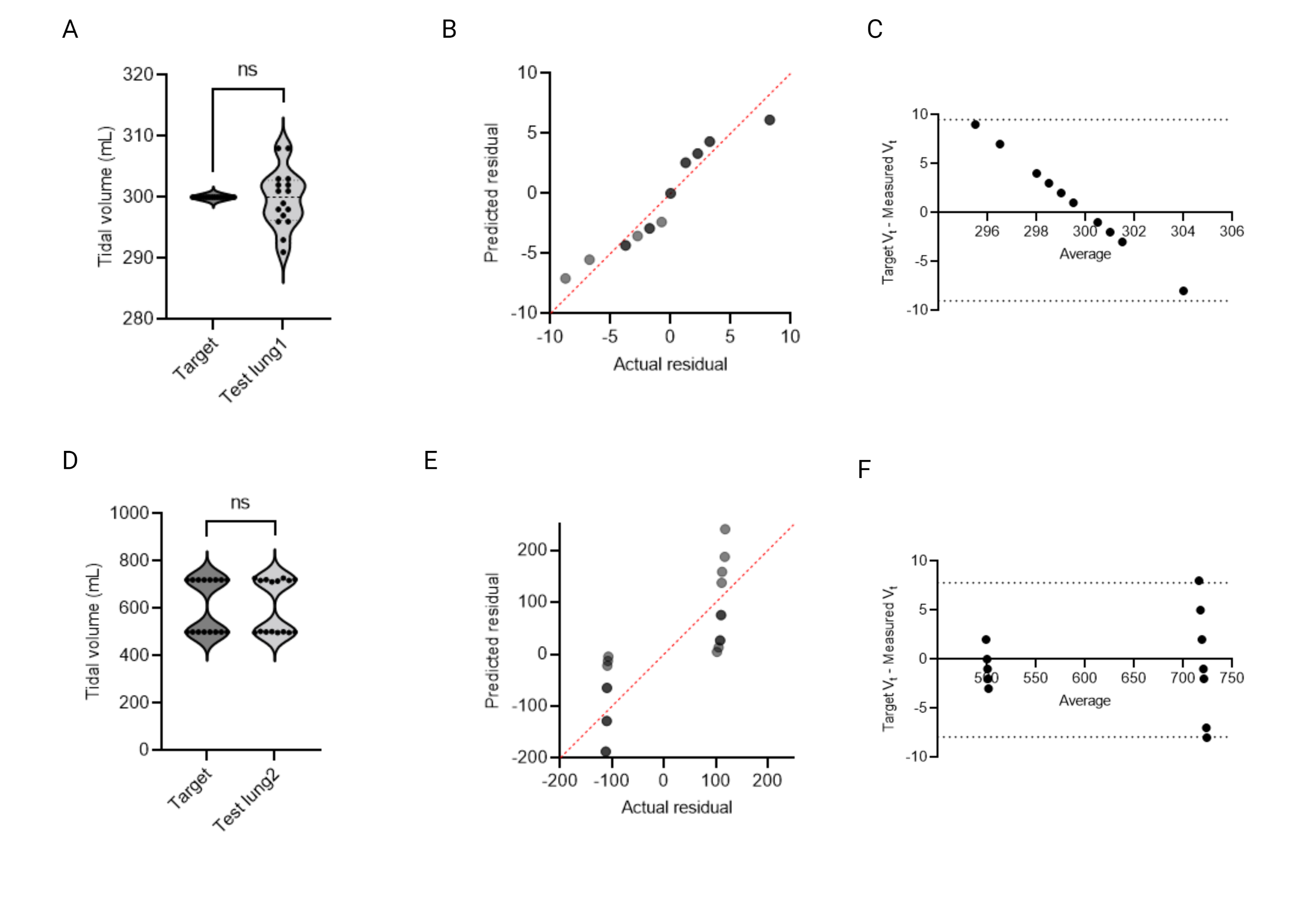


Figure S7. In benchtop testing, a single ventilator was employed to deliver the set tidal volume (V_t_) to two test lungs using VSS. Panels (A-C) and (D-F) show violin plots, quantile-quantile (Q-Q) plots, and Bland-Altman plots in test lungs 1 and 2.

Table S1. **Comparison of performance of VSS with NM3 respiratory profile monitor**. The comparison of the performance of VSS with NM3 respiratory profile monitor in an anesthetized and orotracheally intubated swine under normoxia and ARDS-phenotype conditions was presented. We compared the performance of VSS against an FDA-approved respiratory profile monitor, NM3 (Philips Healthcare, Wallingford, CT, USA). The data were presented from normal healthy conditions and after warm saline lavage-induced ARDS phenotype under different ventilation modes.

(PCV = Pressure Control Ventilation mode; i-time = inspiratory time; RR = respiratory rate; VTi = inspired tidal volume; VTe= expired tidal volume; VTave = average tidal volume; PEEP = positive end expiratory pressure; MeanPaw = mean airway pressure; PIF = peak inspiratory flow; PEF = peak expiratory flow; RR = respiratory rate; T_i_=inspiratory time; T_e_ = expiratory time)

Table S2. **Differential mechanical ventilator sharing in swine under different health conditions**. The ventilator-sharing system was connected to two swine. Individualized mechanical ventilation support was provided while maintaining animals under different health conditions (normoxia and hypoxia (acute respiratory distress syndrome [ARDS]-like injury phenotype caused by warm saline lavage)). The data from four pigs under normoxia and hypoxia conditions were combined. IP = inspiratory pressure; Vti = inspired tidal volume

**Acronyms/abbreviations used in the manuscript**

| **Acronym** | **Definition** | **Units** | **Short Description** |
| --- | --- | --- | --- |
| bpm | Breaths per minute |  |  |
| Crs | Dynamic compliance | mL/cm H_2_O | Compliance of the respiratory system |
| FiO_2_ | Fraction of inspired Oxygen | Unitless or % | Percentage of oxygen in gas delivered to the patient (air = 21%) |
| i.m | intramuscular |  |  |
| IP | Inspiratory Pressure | cm H_2_O | Pressure target in pressure-controlled ventilation |
| i-time | Inspiratory time | s | Time during inhalation |
| IV | Intravenous |  | Method of delivering drugs via venous access |
| MeanPaw | Mean airway pressure | cm H_2_O | Average airway pressure during breathing |
| Paw | Airway Pressure | cm H_2_O | Pressure at the airway or y-piece |
| PCV | Pressure Control Ventilation mode | cm H_2_O | Method of ventilating a patient that provides increased pressure level during inhalation. Can be used to breath for a sedated patient. |
| PEEP | Positive End Inspiratory Pressure | cm H_2_O | Baseline pressure provided to the patient by a ventilator to reduce collapsed alveoli |
| PEF | Peak Expiratory Flow | L/s | Highest flow during exhalation |
| PetCO_2_ | The partial pressure of carbon dioxide in the breath | Mm Hg | Non-invasive measurement of carbon dioxide in the breath |
| PIF | Peak Inspiratory Flow | L/s | Highest flow during inhalation |
| PPE | Personal protective equipment |  | Gowns, masks, and other materials used to protect the clinician from infection |
| PSV | Pressure Support Ventilation | cm H_2_O | Method of ventilating a patient that provides increased pressure level during inhalation. Used to support spontaneous breathing. |
| R | Resistance | cm H_2_O/L/min | Resistance of the respiratory system to flow |
| RR | Respiratory Rate | bpm | Number of breaths per minute |
| SpO_2_ | Oxygen saturation is the fraction of oxygen-saturated haemoglobin relative to total haemoglobin in the blood | % | Non-invasive measurement of oxygen content in the blood using pulse oximeter |
| s.q | subcutaneous |  |  |
| stdev | standard deviation |  |  |
| T_e_ | Expiratory time | s | Time spent during exhalation |
| T_i_ | Inspiratory time | s | Time during inhalation |
| VC | Volume Control ventilation mode |  | Method of ventilating a patient that provides a set volume at every breath. |
| VMS | Ventilator Monitoring System |  | A system for remote monitoring of ventilated patients |
| VSS | Ventilator-Sharing System |  | A system that allows a single ventilator to support multiple patients |
| V_t_ | Tidal Volume | mL | The total volume of a breath |
| VTave | Average tidal volume | mL | Average tidal volume over many breaths |
| VTe | Expired tidal volume | mL | Tidal volume exhaled during a breath |
| VTi | Inspired tidal volume | mL | Tidal volume inhaled during a breath |
